# Genome-wide identification and expression analysis of the *Eriobotrya japonica TIFY* gene family reveals its functional diversity under abiotic stress conditions

**DOI:** 10.1186/s12864-024-10375-2

**Published:** 2024-05-14

**Authors:** Xulin Li, Ke Wen, Ling Zhu, Chaoying Chen, Tuo Yin, Xiuyao Yang, Ke Zhao, Yinqiang Zi, Huiyun Zhang, Xinping Luo, Hanyao Zhang

**Affiliations:** 1 Key Laboratory for Forest Resources Conservation and Utilization in the Southwest Mountains of China, Ministry of Education, Southwest Forestry University, Kunming, 650224 China; 2 Key Laboratory of Biodiversity Conservation in Southwest China, National Forest and Grassland Administration, Southwest Forestry University, Kunming, 650224 China; 3 Institute of Tropical and Subtropical Cash Crops, Yunnan Academy of Agriculture Sciences, Baoshan, 678000 China

**Keywords:** Loquat, *TIFY* gene family, Evolution, Abiotic stress, Expression profile

## Abstract

**Background:**

Plant-specific TIFY proteins are widely found in terrestrial plants and play important roles in plant adversity responses. Although the genome of loquat at the chromosome level has been published, studies on the TIFY family in loquat are lacking. Therefore, the *EjTIFY* gene family was bioinformatically analyzed by constructing a phylogenetic tree, chromosomal localization, gene structure, and adversity expression profiling in this study.

**Results:**

Twenty-six *EjTIFY* genes were identified and categorized into four subfamilies (ZML, JAZ, PPD, and TIFY) based on their structural domains. Twenty-four *EjTIFY* genes were irregularly distributed on 11 of the 17 chromosomes, and the remaining two genes were distributed in fragments. We identified 15 covariate *TIFY* gene pairs in the loquat genome, 13 of which were involved in large-scale interchromosomal segmental duplication events, and two of which were involved in tandem duplication events. Many abiotic stress cis-elements were widely present in the promoter region. Analysis of the Ka/Ks ratio showed that the paralogous homologs of the *EjTIFY* family were mainly subjected to purifying selection. Analysis of the RNA-seq data revealed that a total of five differentially expressed genes (DEGs) were expressed in the shoots under gibberellin treatment, whereas only one gene was significantly differentially expressed in the leaves; under both low-temperature and high-temperature stresses, there were significantly differentially expressed genes, and the *EjJAZ15* gene was significantly upregulated under both low- and high-temperature stress. RNA-seq and qRT-PCR expression analysis under salt stress conditions revealed that *EjJAZ2*, *EjJAZ4*, and *EjJAZ9* responded to salt stress in loquat plants, which promoted resistance to salt stress through the JA pathway. The response model of the *TIFY* genes in the jasmonic acid pathway under salt stress in loquat was systematically summarized.

**Conclusions:**

These results provide a theoretical basis for exploring the characteristics and functions of additional *EjTIFY* genes in the future. This study also provides a theoretical basis for further research on breeding for salt stress resistance in loquat. RT-qPCR analysis revealed that the expression of one of the three *EjTIFY* genes increased and the expression of two decreased under salt stress conditions, suggesting that *EjTIFY* exhibited different expression patterns under salt stress conditions.

**Supplementary Information:**

The online version contains supplementary material available at 10.1186/s12864-024-10375-2.

## Background

The *TIFY* family, first identified in *Arabidopsis thaliana*, is a family of plant-specific genes involved in process regulation, such as growth, development and stress responses, and biosynthesis of secondary metabolites [1, 2]. The *TIFY* family is named after the highly conserved TIFY structural domain, previously known as zinc finger protein expressed in inflorescence meristematic tissues (ZIM) [3, 4]. The *TIFY* gene family can be divided into four subfamilies based on its different structural domain characterization and systematic analysis, e.g., TIFY, PPD, JAZ, and ZML. Among these four subfamilies, the TIFY subfamily contains only TIFY structural domains, while the other subfamilies have specific structural domains in addition to TIFY structural domains [5]; the ZML subfamily of proteins can respond to the DNA-binding C2C2-GATA zinc finger structural domain and a CCT structural domain involved in protein interactions; the JAZ subfamily contains the CCT_2 conserved structural domain; and the PPD subfamily proteins have a unique PPD structural domain at the N-terminal end and a truncated Jas structural domain lacking the conserved PY residue [6, 7].

*TIFY* plays a crucial role in plant growth and development and is widely involved in the developmental processes of organs and tissues such as stems, leaves, and flowers. In particular, the JAZ subfamily can serve as a vital component of the JA signaling pathway, which is involved in plant reproductive development. Upregulation of *AtJAZ7* resulted in early flowering in *A. thaliana* [8]; furthermore, *AtJAZ1*, *AtJAZ8*, and *AtJAZ11*, as well as the targeting of *MYB 21* and *MYB 24*, specifically regulate male fertility [9]. Based on the vital role of the *TIFY* gene family in plant growth and development, the *TIFY* gene family has been characterized in *A. thaliana* [4], *Oryza sativa* [10], *Vitis vinifera* [7], *Malus domestica* [11], *Prunus persica* [2] and *Populus trichocarpa* [12]. In addition, functional information on various TIFY proteins has been obtained, demonstrating their regulatory roles in plant growth and development and their responses to biotic and abiotic stresses [1, 13]. Overexpression of *GsJAZ2* has been shown to enhance salinity tolerance in *Glycine max* [14]; salt-induced overexpression of *OsJAZ8* in early *O. sativa* resulted in greater salt stress tolerance than did wild type during development [15]; overexpression of *AtJAZ1* or *AtJAZ4* reduced the response of *A. thaliana* to extreme temperature stress by modulating the jasmonate signaling pathway [16]; and overexpression of *GsTIFY10* enhanced the tolerance of wild *G. max* to bicarbonate stress [17]. It has been predicted that in the future, the timing and relative intensity of abiotic stresses will become more unpredictable and extreme [18]. In addition, the impact of multiple stresses (heat and salt) on plant growth and development is expected to increase significantly under the influence of climate change [19]. Although the use of combined bioinformatic and transcriptomic approaches is more conducive to exploring the mechanisms of plants in response to abiotic stresses, it is particularly vital to reveal the mechanisms of the loquat *TIFY* gene family in response to abiotic stresses.

Loquat *(Eriobotrya japonica)* is a plant of the Rosaceae family originating from subtropical China. It is an evergreen tree species, and the planted area and total production of *E. japonica* in China are high [20]. Its fruit is delicious, popular, and has traditional medical effects. The fruits and leaves of *E. japonica* are the parts with the highest economic value, and these fruits are used for making drinks, jams, and preserved fruits, in addition to direct consumption; the leaves of this plant can be utilized as a traditional Chinese herbal medicine, which has the effects of cleaning the lungs and stomach, relieving coughs and resolving phlegm [21]. Nevertheless, the growth and development of *E. japonica* are sensitive to changes in environmental factors such as temperature, salt, and gibberellin, which leads to a decrease in the yield and quality of *E. japonica* fruits. Although the current studies on the genetic aspects of *E. japonica* have focused mainly on the expression regulation and functional analysis of the genes regulating the flowering time of triploid *E. japonica* and these studies identified the genes involved in the control of *E. japonica* flowering time [22], the study of the *E. japonica TIFY* gene family under salt stress has not yet provided any relevant reports. Therefore, it is vital to study the regulatory mechanisms of *E. japonica* under abiotic stress to maintain its economic value. In this study, we identified 26 *EjTIFY* genes categorized into four subfamilies. Comprehensive analyses of gene structure, motif composition, cis-acting elements in promoters, chromosomal distribution, gene duplication, and phylogenetic and covariate relationships were completed. We also analyzed the expression profiles of three *TIFY* genes (*EjJAZ2*, *EjJAZ4*, and *EjJAZ9*) among the genes related to salt treatment-related DEGs in the leaves of *E. japonica* under temperature and salt stress conditions via RNA-seq. These three genes were also shown to be differentially expressed in the jasmonate pathway, and qRT-PCR expression analysis of three salt stress-responsive genes was performed. The results of this paper can provide valuable information for future functional analysis of the *TIFY* gene family.

## Results

### Identification of the *EjTIFY* gene family and analysis of its physicochemical properties

**Fig. 1:**
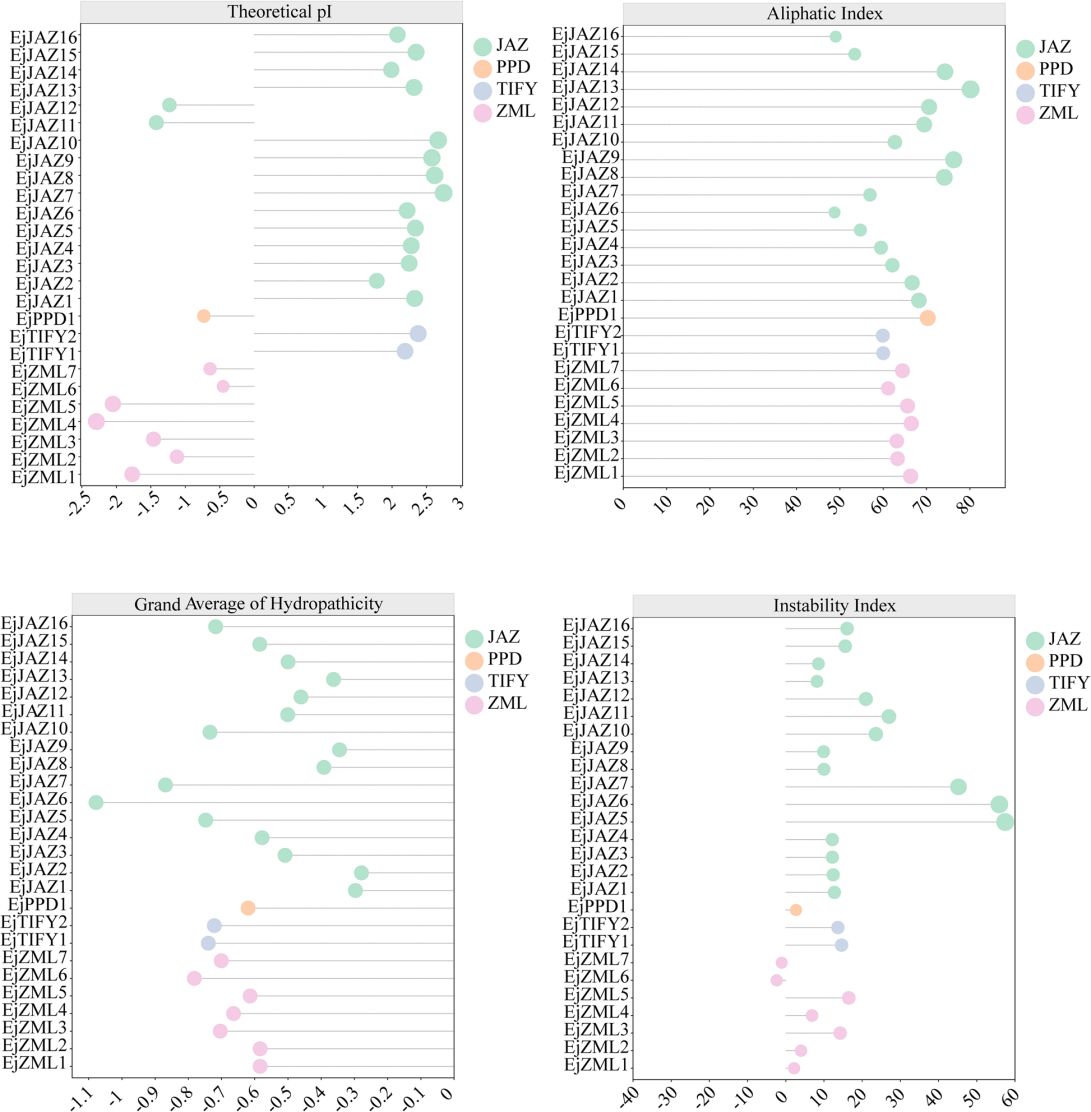
Physicochemical property analysis of *EjTIFY* family members. The isoelectric point, lipolysis index, hydrophilic index, and instability coefficient of the *EjTIFY* genes are indicated in the figure

A total of 26 *TIFY* genes were identified in the whole genome of *E. japonica* via BLAST and HMMER searches. The conserved structural domains in their coding protein sequences were further examined using a CDD search of the NCBI database. The 26 *TIFYs* were categorized into four subfamilies based on motif and structural domain composition. Two proteins with only one TIFY structural domain belonged to the TIFY subfamily and were named EjTIFY1 and EjTIFY2. The remaining 24 proteins had Jas motifs in addition to the TIFY structural domain. Of these Jas motif-containing proteins, 16 proteins with only TIFY structural domains and Jas motifs belong to the JAZ subfamily and are named EjJAZ1 to EjJAZ16. One of the remaining proteins has no P or Y residues in the Jas motif, which makes them identical to the PDD subfamily; this protein is named EjPPDl and contains TIFY structural domains, a GATA zinc finger structural domain, a Jas motif, and a CCT motif. The remaining seven proteins belonged to the ZML subfamily and were named EjZML1 to EjZML7. We identified 16 JAZ, 1 PPD, 2 TIFY, and 7 ZML proteins in *E. japonicas* (Table S2). The results of the physicochemical property analysis of these 26 proteins showed (Fig. 1) that the number of amino acids in the EjTIFY protein varied from 114 aa (EjJAZ5) to 445 aa (EjTIFY2). The predicted molecular weights of EjTIFYs also highly changed, within the range of 13.05 kDa (EjJAZ5) to 47.22 kDa (EjTIFY2). Sixteen of the 26 EjTIFYs had theoretical PI values greater than 7, indicating that most TIFY proteins in *E. japonica* are fundamental. Subcellular localization analysis (Table S2) revealed that the remaining 22 proteins were located in the nucleus, except for EjJAZ11 and EjJAZ14, which are located on chromosomes; EjPPD1, which is located in the mitochondria; and EjZML2, which is located in the *Golgi apparatus*.

### Phylogenetic analysis

**Fig. 2:**
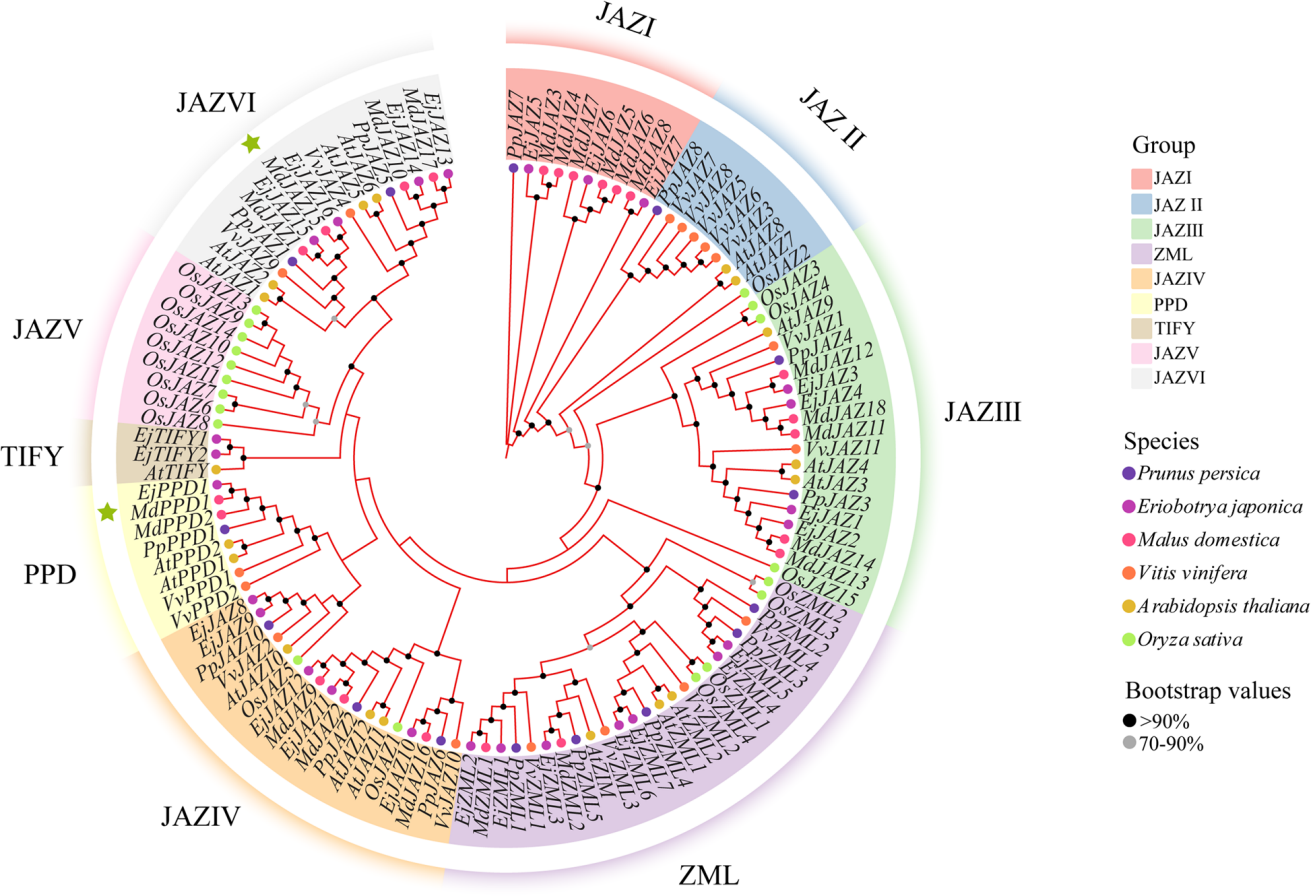
Phylogenetic relationships of the TIFY proteins of *E. japonica*, *A. thaliana*, *O. sativa* (*O. sativa*), *V. vinifera*, *M. domestica*, and *P. persica*, with the green pentagrams indicating that the relevant function of the gene has been demonstrated

The TIFY protein sequences of *E. japonica, A. thaliana*, *O. sativa*, *V. vinifera*, *M. domestica*, and *P. persica* were aligned with each other via MAFFT software to clarify the evolutionary relationships between the *EjTIFY* gene family and other species. Multiple TIFY protein sequences were compared, and a phylogenetic tree was constructed using the maximum likelihood method. Based on the results of the phylogenetic tree and the classification of TIFY proteins in other species [23–25], the TIFY proteins were categorized into nine subfamilies, namely, JAZ I-VI, PPD, TIFY, and ZML (Fig. 2). Among them, the JAZ subfamily had the greatest number of proteins, including 12 AtJAZ proteins, 16 EjJAZ proteins, 18 MdJAZ proteins, 15 OsJAZ proteins, 10 PpJAZ proteins, and 11 VvJAZ proteins. The TIFY subfamily had the lowest number of genes, with only 2 EjTIFY and 1 AtTIFY protein. No monocotyledons were in the PPD or TIFY subfamilies, consistent with previous studies [7]. There were two AtPPD proteins, two VvPPD proteins, one EjPPD protein, two MdPPD proteins, and one PpPPD protein in the PPD subfamily and one AtTIFY protein and two EjTIFY proteins in the TIFY subfamily. Further analysis revealed that the number of TIFY genes in *E. japonica* and *M. domestica* was greater than that in *A. thaliana*. This may be related to the fact that *E. japonica* and *M. domestica* have undergone more fusion and duplication events during the evolutionary process [26]. As a dicotyledonous plant, *E. japonica* is more closely related to *A. thaliana*, *V. vinifera*, *P. persica*, and *M. domestica*. Based on the evolutionary tree showing that *E. japonica* and dicotyledonous plants (*M. domestica*) have a significantly greater number of clusters on the same branch than other species, it can be further inferred that *E. japonica* is more closely related to the dicotyledonous plant *M. domestica*.

### Conserved motif analysis and gene structure analysis

**Fig. 3:**
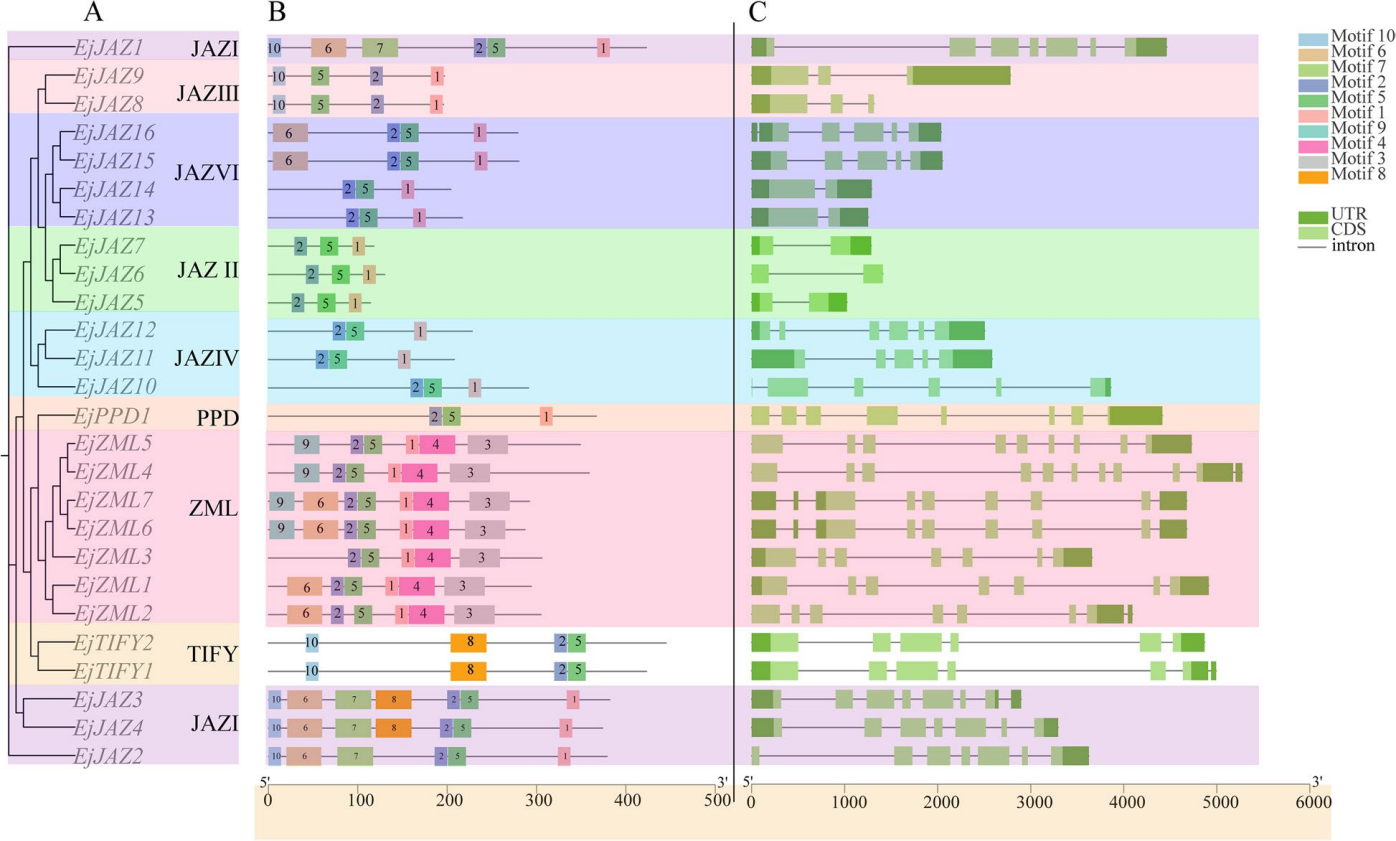
*EjTIFY* evolutionary tree (**A**), predicted conserved motifs (**B**) and gene structure (**C**)

To further reveal the structural and functional features of *EjTIFY*, the conserved structural motifs of the EjTIFY protein were analyzed using the online website MEME. Ten conserved motifs in the *E. japonica* JAZ, PPD, TIFY, and ZML proteins were identified. The results show (Fig. 3B) that the number of motifs in JAZ, PPD, TIFY, and ZML members varied from 3 to 7. All members of the *E. japonica* JAZ, PPD, TIFY, and ZML proteins shared motif 2 and motif 5. In addition, certain motifs were restricted to specific subfamilies, such as motif 3 and motif 4, which belong to the ZML subfamily, and motif 7, which is included in the JAZ I group. In general, members of the same subfamily or group usually have the same type and number of motifs, and the distribution of motifs is usually the same. For example, members of the TIFY subfamily contain four main motifs, members of the PPD subfamily have three main motifs, members of the JAZ I subfamily have seven major motifs, members of both the JAZ II and JAZ IV subfamilies have three main motifs, and members of the JAZ III subfamily contain mainly four motifs, and three to four motifs are included in the JAZ VI subfamily.

Exon-intron structure plays a vital role in the evolution of gene families and can be used to support phylogenetic grouping [27]. Our results revealed (Fig. 3C) some differences in exon number and intron length among TIFY gene family members. For example, the number of exons ranged from 2 to 10, whereas *EjZML4* had ten exons, which was much greater than that of other TIFY genes. The exon/intron organization of genes also suggests that genes in the same subfamily/group have fewer structural differences and tend to have similar gene structures and exon/intron numbers. In the JAZ subfamily, the 12 *EjJAZ* genes had 3 to 6 exons, except for the *EjJAZ9*, *EjJAZ8*, *EjJAZ7*, *EjJAZ6*, and *EjJAZ5* genes, which had only two exons. All seven genes in the ZML subfamily have 5 to 9 exons. This finding suggested that many TIFY genes are conserved, whereas several genes, *EjJAZ9*, *EjJAZ8*, *EjJAZ7*, *EjJAZ6*, and *EjJAZ5*, have only two exons, and it is hypothesized that these genes may have lost their exons during evolution [28].

### Analysis of promoter cis-acting elements

**Fig. 4:**
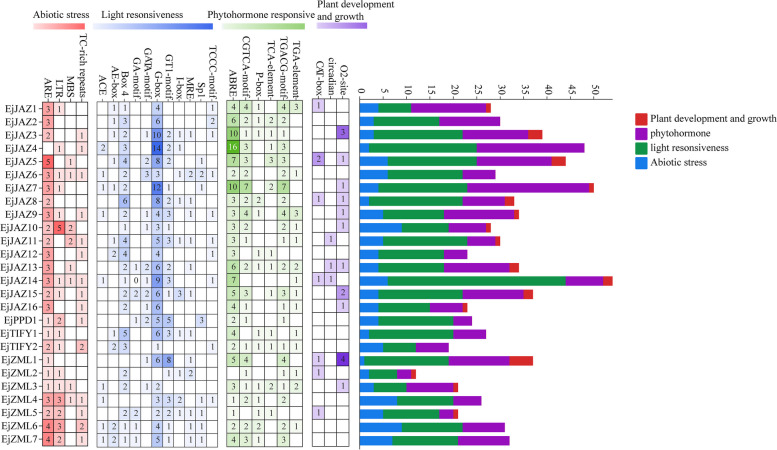
Cis-acting elements of the *EjTIFY* gene. The numbers and different color gradients indicate the number of cis-acting elements. The color-coded histograms represent the number of cis-acting elements for each gene classified into four categories according to functional factors: light response, phytohormone response, abiotic stress, and plant growth and development

To further elucidate the potential functions of *EjTIFY* gene family members, cis-acting elements in the 2,000 bp regions upstream of the promoter were analyzed. The results showed (Fig. 4) that *EjTIFY* members contain four types of cis-acting elements, e.g., light-responsive, hormone-responsive, defense- and stress-responsive, and growth- and development-related elements. Light-responsive elements are present in all *EjTIFY* members, accounting for a high proportion. It is hypothesized that *EjTIFY* members are closely related to light regulation in *E. japonica*. Eight elements related to hormone signaling pathways were identified: the TGA element, the TATC box, the TCA element, the P box, the ABRE, the AuxRR core, the CGTCA motif, and the TGACG motif. These homeopathic-acting elements are involved in growth hormone, gibberellin, salicylic acid, abscisic acid, and methyl jasmonate metabolic regulation. All 22 *EjTIFYs*, except *EjZML5*, *EjTIFY1*, *EjJAZ14*, and *EjJAZ12*, contained methyl jasmonate response elements (TGACG motif and CGTCA motif). All 25 *EjTIFYs* had ABRE response elements except *EjZML2*, which did not contain ABRE response elements, indicating that most *EjTIFYs* can participate in Me-, JA-, and ABA-mediated signaling pathways. Four abiotic stress-related action elements were also identified, namely, low-temperature response elements (LTRs), drought-inducible elements (MBSs), anaerobic-inducibles (AREs), and defense- and stress-responsive elements (TC-rich repeats), among which *EjJAZ10* contained the most low-temperature response elements; thus, we can speculate that this gene may be highly expressed under low-temperature stress. Among the drought-inducing elements, the *EjJAZ10* and *EjJAZ11* genes were the most abundant.

### Chromosomal distribution and gene duplication analysis of the *EjTIFY* gene

**Fig. 5:**
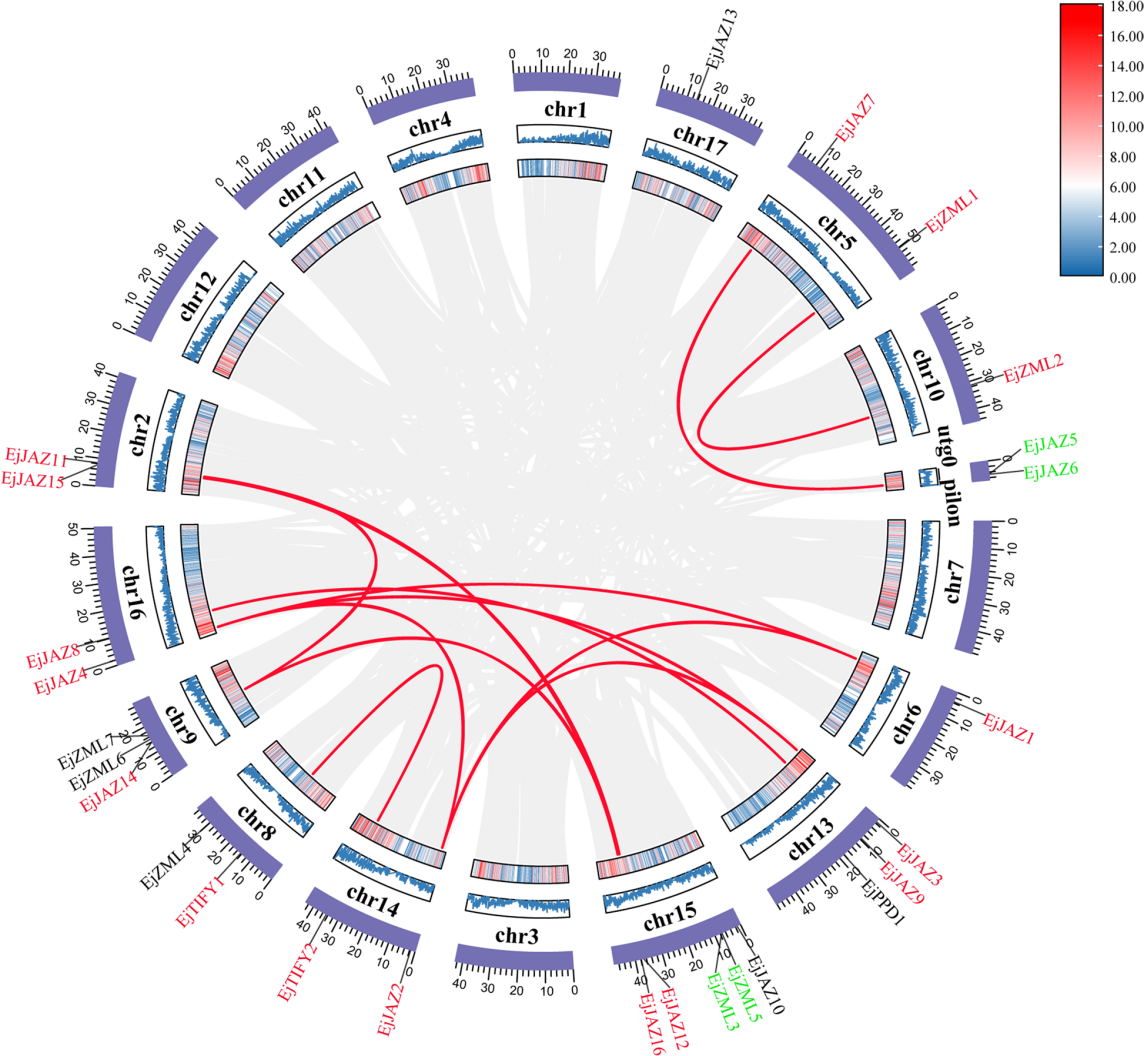
Distribution and covariance of *EjTIFY* on *E. japonica* genomic chromosomes. Those labeled in red and green have covariance, where the red-labeled *EjTIFY* had segmental duplication events, the green-labeled *EjTIFY* had tandem duplication events, and the black-labeled *EjTIFY* lacked covariance. The two rings in the center represent the gene density per chromosome. The red color indicates the covariance between *EjTIFYs*.

The genes were mapped to the genome using the TBtools tool to visualize the chromosomal distribution of the *EjTIFY* genes. Of the 26 *EjTIFY* genes, 24 had 11 chromosomes, and two had the utg0-pilon fragment (Fig. 5). Chromosome chr15 contained the most genes (5). However, chromosomes chr6, chr7, and chr10 had the fewest genes, containing only one gene. Chromosomes chr5, chr14, chr8, chr2, and chr16 contained two genes each, and chromosomes chr9 and chr13 contained three genes each. Notably, the *EjJAZ5* and *EjJAZ6* genes were not on the chromosomes. Combined with previous subcellular localization analyses, both genes were inside the nucleus, and it was hypothesized that these genes might be caused by gene annotation errors; however, this requires further experimental verification.

To further determine the amplification and evolution of the *EjTIFY* genes, the *E. japonica* gene duplication events were analyzed using MCScanX, and a total of 15 duplication pairs were identified (Fig. 5) (13 fragment duplication gene pairs and two tandem duplication gene pairs). Notably, one tandem duplicate gene pair did not exist on the chromosome and existed within the segment. These results suggest that *EjTIFY* members may expand through tandem and segmental duplication events; in particular, segmental duplications may be the prime driver of the expansion of the *EjTIFY* gene family. Ka/Ks ratios can be used to elucidate the evolutionary processes and selective pressures on *EjTIFY* [29], so we calculated the Ka/Ks ratios of all 15 pairs of genes (Table S3). All 15 pairs of duplicated genes had Ka/Ks ratios less than 1, suggesting that the *EjTIFY* gene pairs may have undergone purifying selection during the evolutionary process and played a vital role in maintaining the conserved structure of the *EjTIFY* genes [30].

### Cotemporal and evolutionary analysis of *EjTIFY* genes with *TIFY* genes from other plants

**Fig. 6:**
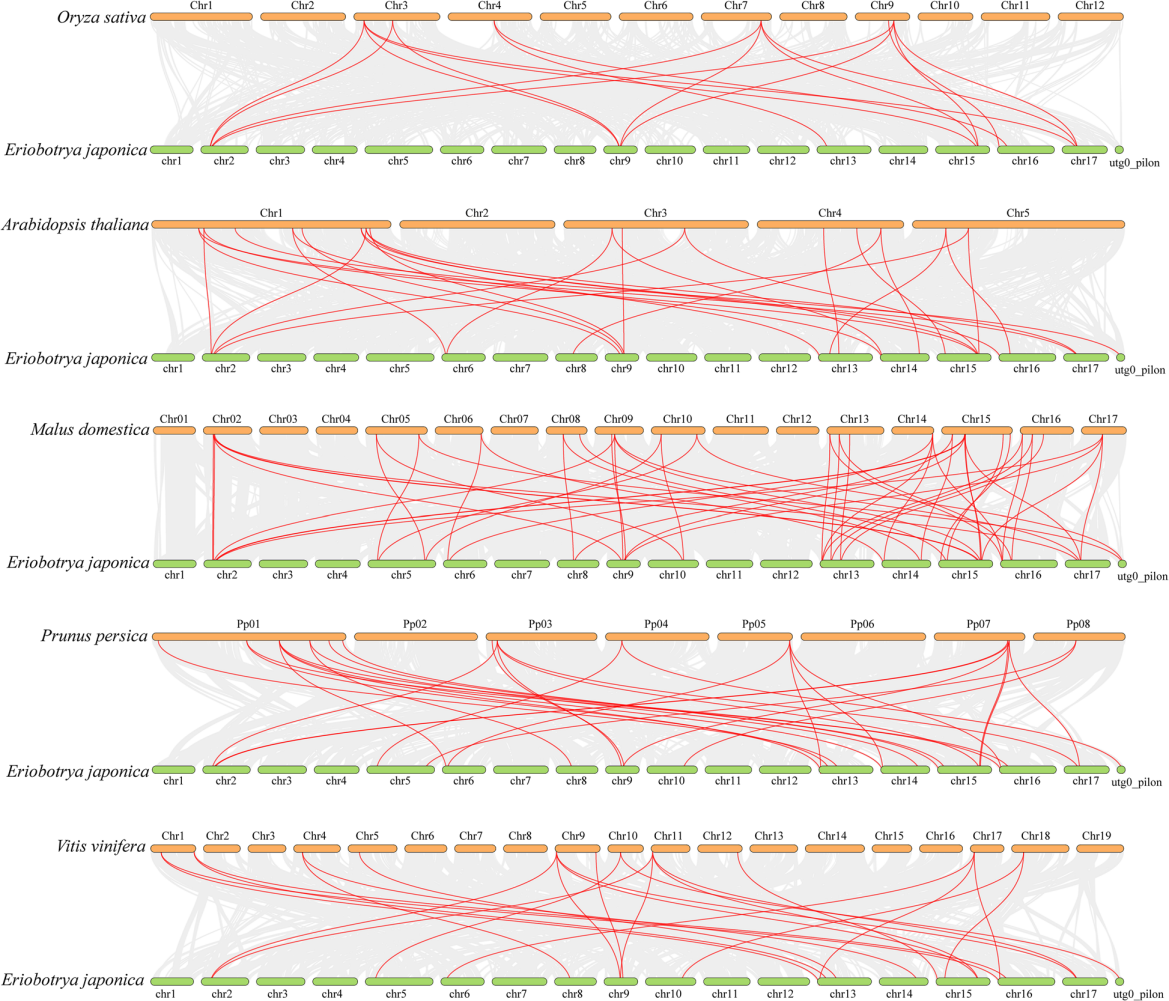
Collinearity analysis of *TIFY* genes in *E. japonica* and five representative plants. The gray line in the background indicates the colinear blocks in *E. japonica* and other plant genomes, while the red line highlights the colinear *TIFY* gene pairs

Colineage analysis among different species is a method for studying their evolution and affinities [31]. To further explore the gene duplication time of *EjTIFY* and infer its phylogenetic mechanism, we chose five representative species for comparative analysis of covariance with *E. japonica*, including four dicotyledons (*A. thaliana*, *M. domestica*, *P. persica*, and *V. vinifera*) and one monocotyledon (*O. sativa*) (Fig. 6). The homology of *E. japonica* with *M. domestica* was 52 pairs, followed by *P. persica* [32], *A. thaliana* [26], *V. vinifera* [7], and *O. sativa* [16]. The results showed that the covariance between the *E. japonica* and dicotyledonous plant genomes was greater than that between the monocotyledonous plant genomes. Individual homologous genes of one-to-many or many-to-one purity were identified. Among them, seven *EjTIFY* genes (*EjJAZ16*, *EjJAZ13*, *EjJAZ15*, *EjJAZ14*, *EjJAZ9*, *EjJAZ8*, and *EjJAZ4*) showed covariant associations among all five representative species, suggesting that these genes in the *EjTIFY* gene family played a vital role in the evolutionary process [33].

### Expression analysis of the *EjTIFY* gene under gibberellin treatment

**Fig. 7:**
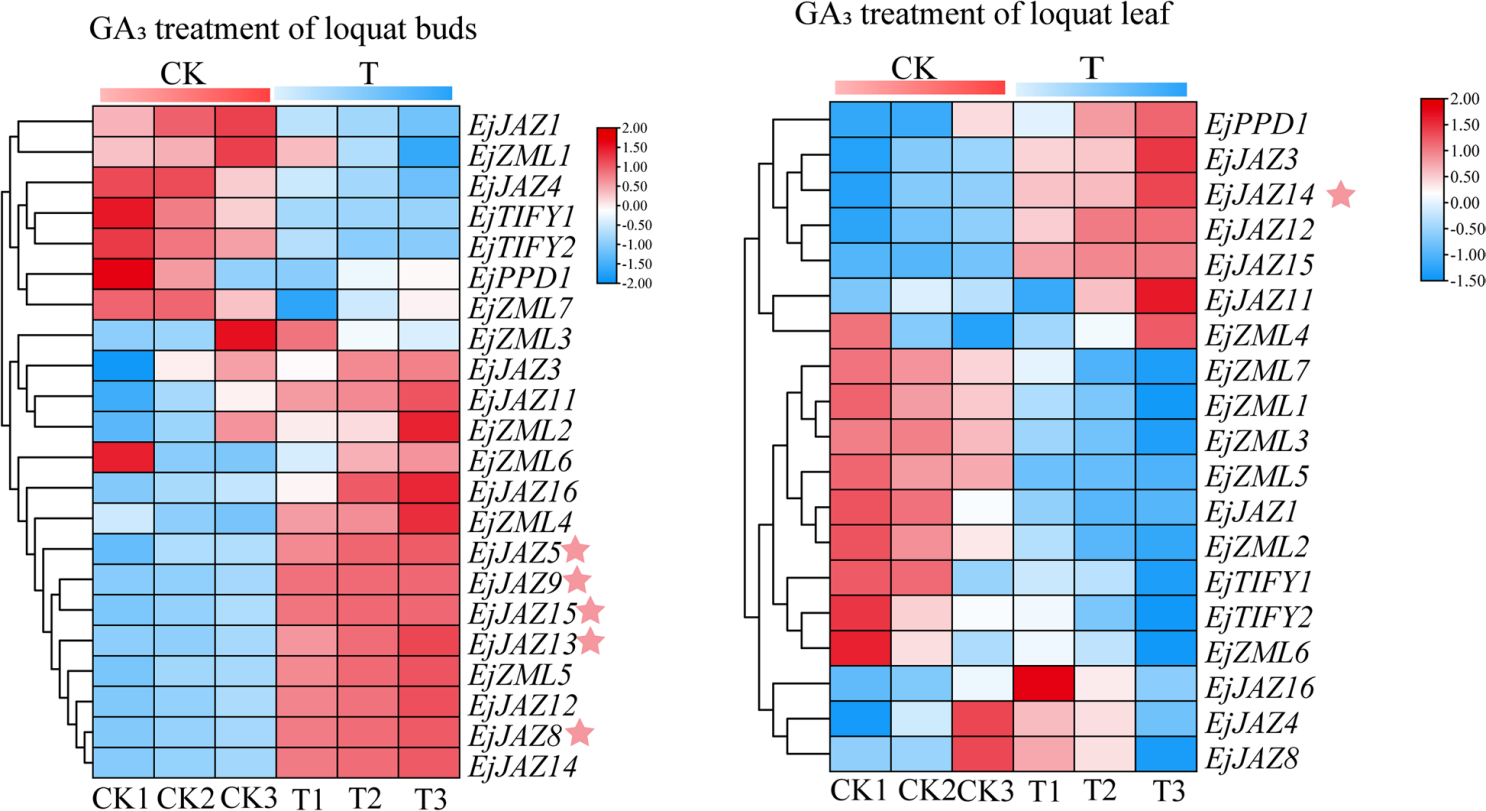
Expression analysis of *E. japonica* buds and leaves under GA_3_ treatment. The pink stars in the figure indicate significantly differentially expressed upregulated genes

The transcriptome data of the GA_3_ treatment and control groups were analyzed for expression using *E. japonica* buds and leaves as materials to explore the response of *E. japonica* to the GA_3_ hormone. The results showed (Fig. 7) that 22 *EjTIFY* genes were expressed in buds, and 19 *EjTIFY* genes were expressed in leaves. In addition, the DEGs of the *EjTIFY* genes in the leaves and shoots in the GA_3_ treatment group were also analyzed. There were five significantly differentially expressed genes in the shoots whose expression was upregulated. However, there was only one significantly differentially expressed gene in the leaves, indicating that members of the *EjTIFY* gene family tended to be more highly expressed in the buds than in the leaves.

### Expression analysis of* the EjTIFY* genes under abiotic stresses

**Fig. 8:**
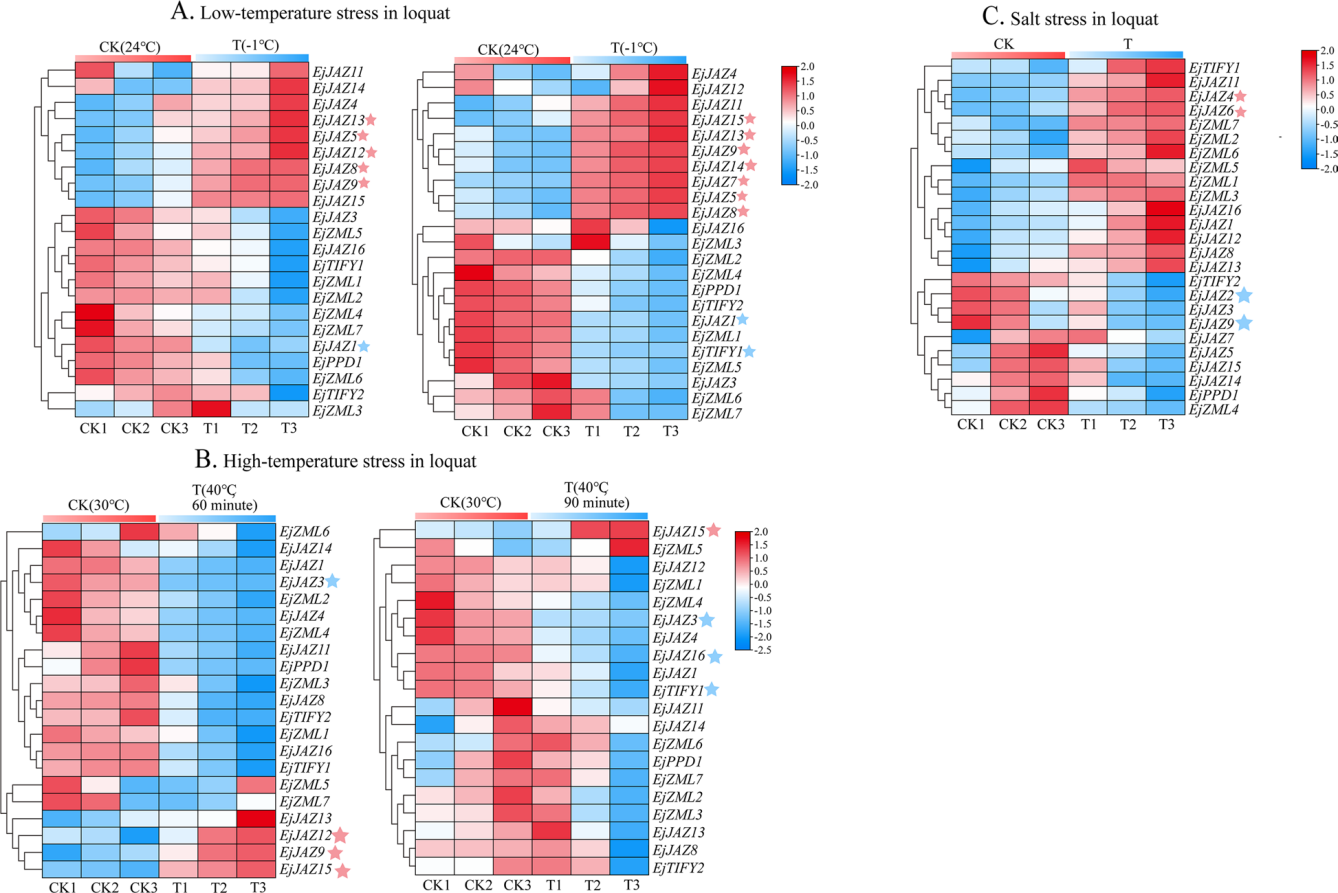
Expression analysis of *E. japonica* under abiotic stress. **A**, **B**, and **C** show the expression profiles of *E. japonica* under low temperature, high temperature, and salt stress, respectively. The pink and blue pentagrams in the figure indicate upregulated and downregulated genes, respectively

To study the relative expression levels of the *EjTIFY* family genes and better understand the functions of the *EjTIFY* family genes, we analyzed the transcriptome data of *E. japonica* under salt, low-temperature, and high-temperature stress. Heatmaps of their expression patterns were constructed using log_2_(TPM + 1) values, and the results (Fig. 8) showed that analysis of the *EjTIFY* family indicated that most genes were upregulated and downregulated in response to low-temperature, high-temperature, and salt stresses. We found that the *EjJAZ* genes were highly upregulated under the high-temperature, salt, and low-temperature treatments. However, the expression of the *EjPPD*, *EjTIFY*, and *EjZML* genes was only slightly induced under the same stresses. Interestingly, the expression of *EjJAZ15* was always significantly upregulated under all the temperature treatments, which may be worthy of further investigation. Whereas four genes were significantly differentially expressed under salt stress, three genes (*EjJAZ2*, *EjJAZ4*, and *EjJAZ9*) were also significantly differentially expressed under the jasmonate pathway. Therefore, the next step was to validate these three genes by real-time fluorescence quantitative RT-PCR analysis.

Analysis of the low-temperature expression data showed (Fig. 8A) that four genes (*EjJAZ8*, *EjJAZ9*, *EjJAZ13*, and *EjJAZ15*) were upregulated in both treatment groups at both temperatures; *EjJAZ1* was downregulated in both treatment groups at both treatments; and all four DEGs and one DEG reached their highest levels at -3 °C. However, up- and down-regulation differences were observed for the remaining genes at both temperatures. For example, *EjJAZ7* was significantly upregulated at -3 °C, but not at -1 °C; *EjTIFY1* was significantly downregulated at -3 °C but was not expressed at -1 °C, suggesting that the *EjJAZ7* and *EjTIFY1* genes are more highly expressed at -3 °C than at -1 °C. The expression level of *EjJAZ15* was relatively more variable among all the genes.

Analysis of *E. japonica* expression under high-temperature stress showed (Fig. 8B) that three genes were differentially and significantly upregulated under high-temperature stress, and three genes were differentially and significantly downregulated under high-temperature stress. *EjJAZ15* was upregulated, and *EjJAZ3* was downregulated in expression in both periods. Notably, we found that the *EjJAZ15* gene was significantly upregulated and expressed under both low- and high-temperature stress, so we can speculate that *EjJAZ15* may be a vital gene affecting *E. japonica* under high-temperature stress.

The analysis of the salt stress expression data showed (Fig. 8C) that most of the JAZ subfamily genes were upregulated under salt stress. However, those genes in other subfamilies showed only lower expression levels. *EjJAZ4* and *EjJAZ6* were differentially and significantly upregulated under salt stress, while *EjJAZ2* and *EjJAZ9* were differentially and significantly downregulated under salt stress. These findings indicate that these four genes may play different roles under salt stress. By analyzing the expression profiles of *EjTIFY* family members in *E. japonica* under low temperature, high temperature, salt, and gibberellin treatments, we found that the JAZ subfamily genes played more vital roles under these stresses than did the other subfamilies.

### GO functional enrichment and pathway analysis

**Fig. 9:**
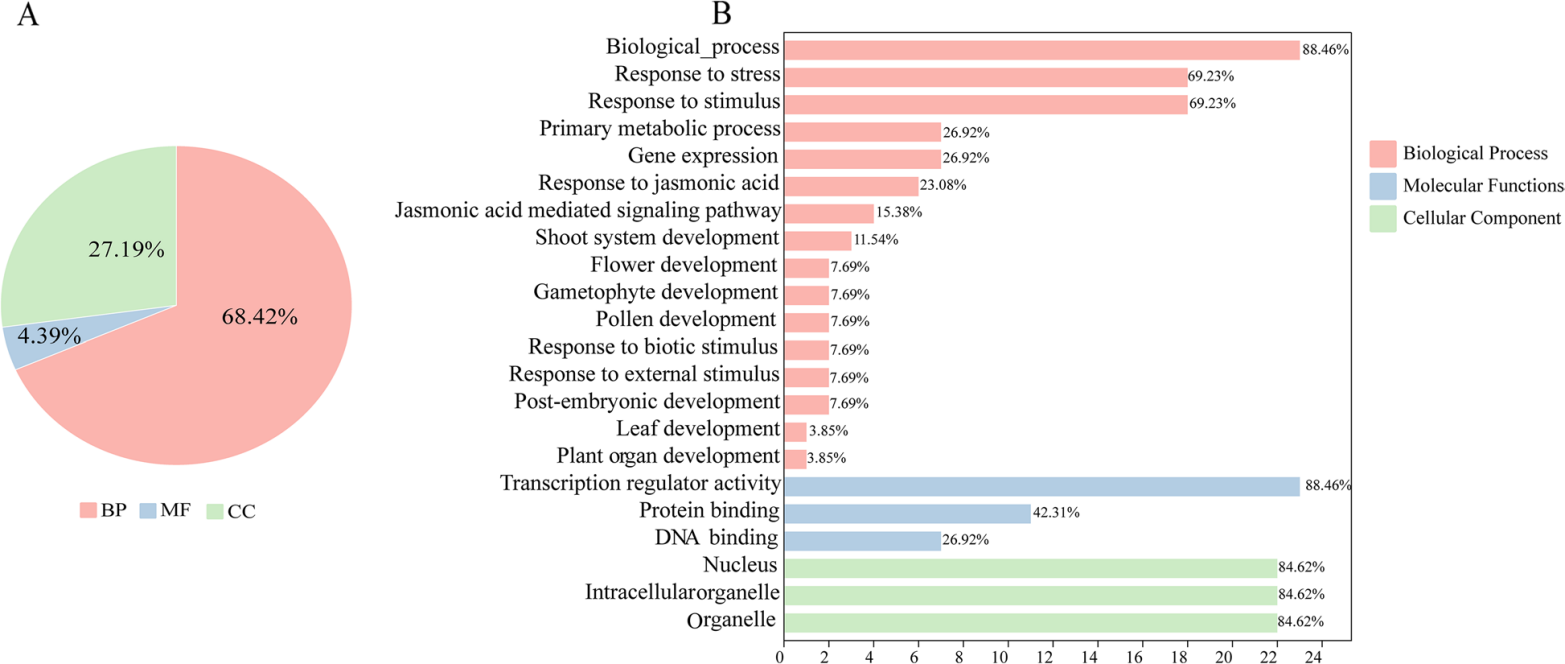
GO enrichment analysis of 23 *EjTIFY* genes under salt stress. A indicates the proportions of the biological processes, molecular functions, and cellular components; B represents the amount of prime GO enrichment

GO functional annotation of genes enables us to better understand the molecular functions of proteins [34], so we utilized the EggNog website for GO annotation analysis (Fig. 9). The 23 EjTIFY proteins were classified into 115 functional groups based on the similarity of protein sequences and categorized into three major groups: biological processes (68.42%), molecular functions (27.19%), and cellular components (4.39%) (Supplementary Table, Fig. 9A). Among the biological processes, *EjTIFY* members were involved in biological processes (88.46%), followed by responses to stress and stimuli (69.23%), major metabolic processes (26.92%), jasmonic acid-mediated signaling pathways (15.38%), and the development of plant organs (3.85%) (Fig. 9B). In addition, both *EjJAZ1*5 and *EjJAZ16* are involved in the response to stimuli to flower, gametophyte, postembryo, and root development, and we can hypothesize that these two genes may play vital roles in the reproductive process of plants. Among the molecular functions, we analyzed more than 88.46% of the annotated proteins with transcriptional regulatory activity, followed by protein binding (42.31%) and DNA binding (26.92%). Annotation of cellular components showed that proteins were annotated as intracellular, nucleus, and organelle in equal percentages, at 84.62%.

**Fig. 10:**
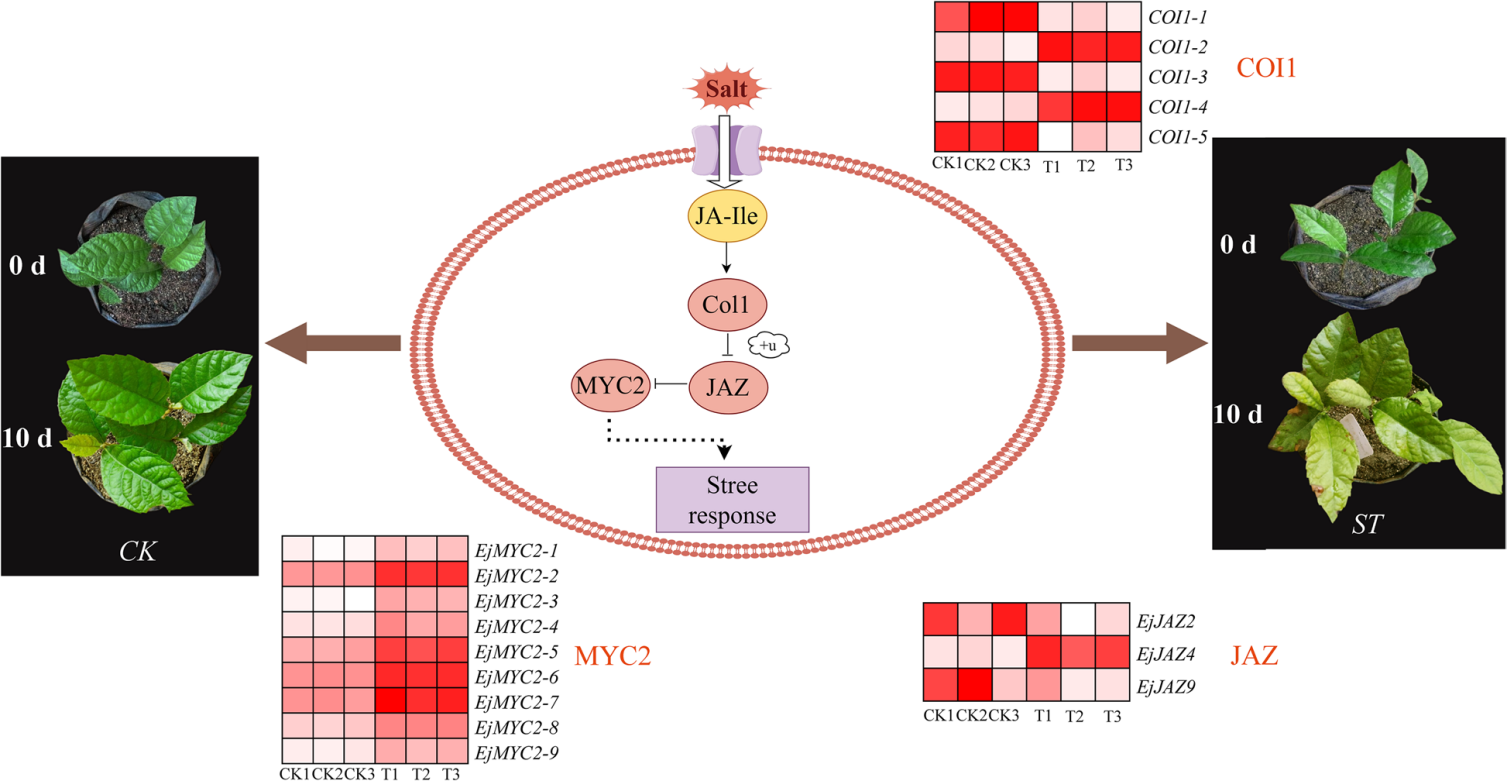
Signal transduction pathway of jasmonic acid under salt stress. The heatmaps in the figure represent the significantly differentially expressed genes for each vital substance under salt stress. This image was drawn by Figdraw

In addition, the KEGG enrichment results showed (Fig. 10) that these genes were enriched only in the jasmonate signaling pathway for phytohormones. JA is an endogenous growth-regulating substance, and when stimulated by the environment or needed for growth, plants regulate the expression of downstream genes through the JA signaling pathway, which balances the processes of plant growth and development and defense responses [35]. First, α-linolenic acid forms jasmonic acid (JA) through a series of transformations in chloroplasts and peroxisomes, and JA forms JA-Ile under the action of the adenylate-forming enzyme of ATP, JAR1 (jasmonic acid resistant 1) [35]. It has been shown (M38) that in the presence of JA-Ile, COI1 can directly bind to the genes in JAZ, so we can hypothesize that three genes, *EjJAZ2*, *EjJAZ4*, and *EjJAZ9*, can also directly bind to COI1 to degrade JAZ in the presence of the 26 S proteasome [36] to relieve the inhibitory effect on MYC2, thus initiating the transcription of JA-responsive genes and improving the ability of *E. japonica* plants to resist salt stress.

### Expression analysis of *EjTIFYS* under salt stress

**Fig. 11:**
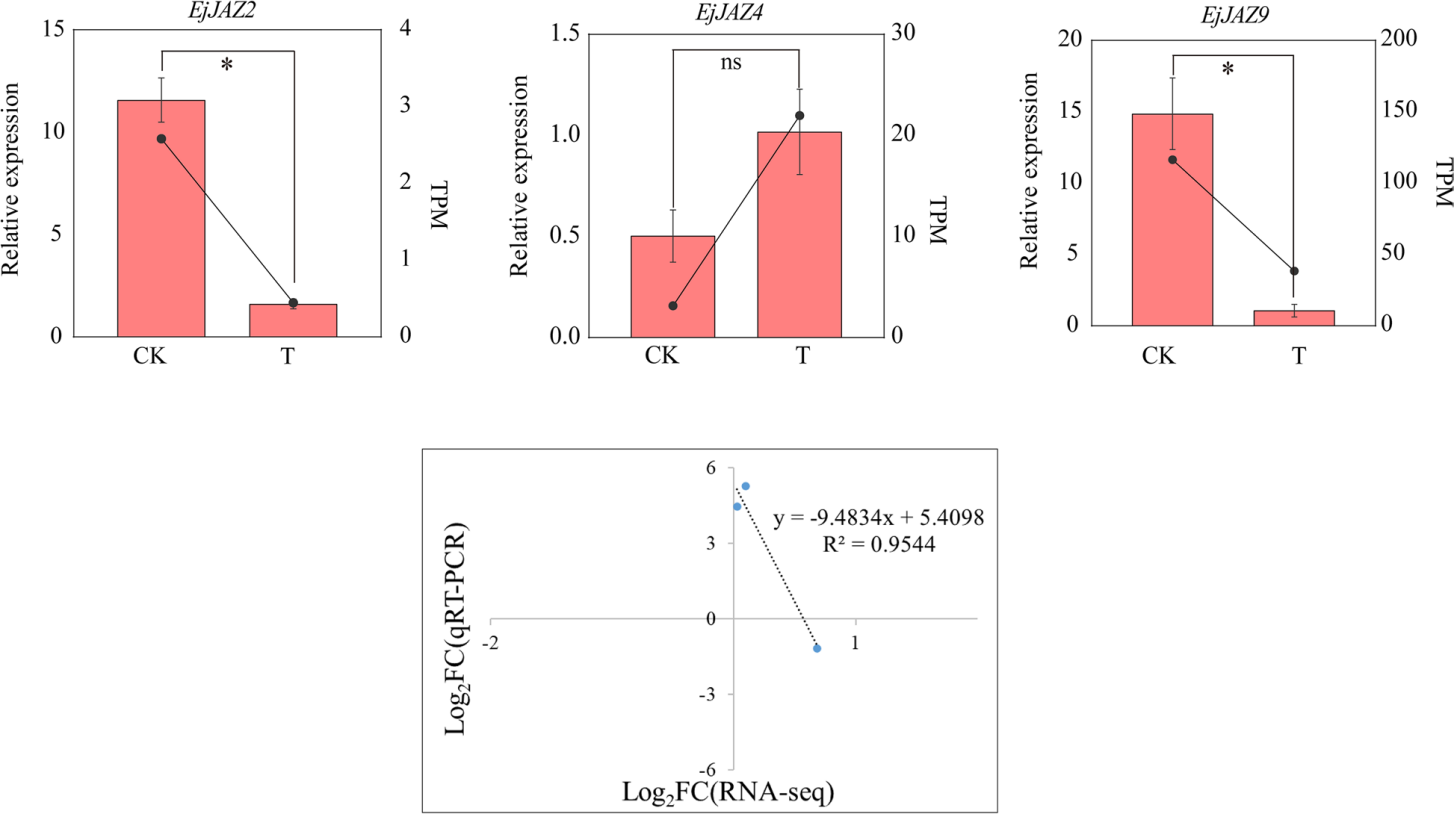
Expression analysis of the *EjTIFY* genes under salt stress. The two dots indicate the RNA-seq expression of the gene, and the pink bar indicates the qRT-PCR expression. Independent sample t-tests were performed using SPSS; ns *p* > 0.05 indicated that the difference was not significant; * *p* < 0.05 indicated that the difference was significant, and each treatment was repeated three times

We selected three differentially and significantly DEGs (*EjJAZ2*, *EjJAZ4*, and *EjJAZ9*) and verified their expression by qRT-PCR analysis to further validate the salt response candidate genes. Figure 11 showed that these genes exhibited completely different expression profiles under salt stress, but their expression trends were consistent with the results of RNA-seq analysis (R^2^ = 0.9544). *EjJAZ4* expression was upregulated under salt stress, while *EjJAZ9* and *EjJAZ*2 expression was downregulated under salt stress, indicating that these genes play a role in the rapid response to salt stress. The different expression patterns of these *EjTIFY* genes suggested that they play various roles in the salt stress response.

## Discussion

The *TIFY* family is a vital family of plant-specific genes and has been characterized in several species of monocotyledons and dicotyledons [4, 10]. It has been demonstrated that members of the *TIFY* gene family, especially the JAZ subfamily, play vital roles in different plant developmental processes and defenses against biotic and abiotic stresses, such as seed germination [37], floral organ development [38], and responses to abiotic stresses, such as salt, high temperature, drought, and biotic stresses of plant diseases [39–41]. However, few functional studies on the *TIFY* gene family members of *E. japonica* have been reported; therefore, in this study, the *TIFY* gene family members were identified in the *E. japonica* genome, and the sequence information of each member and its expression pattern under abiotic stresses were analyzed.

Twenty-six *TIFY* genes were identified in the *E. japonica* genome in this study, and the location of the EjTIFY proteins could be predicted by subcellular localization, except for EjJAZ11 and EjJAZ14, which are located in chloroplasts; EjPPD1, located in the mitochondria; and EjZML2, located in the Golgi apparatus. The remaining 22 TIFY proteins were distributed in the nucleus, suggesting that the *TIFY* gene family developed in plant cells [42]. Notably, the *TIFY* family is present only in terrestrial plants and not in green or nonphotosynthetic eukaryotes [6], suggesting that plant TIFY homologs evolved after aquatic plants adapted to land life [43].

The *TIFY* gene family can be categorized into four subfamilies based on their different structural domains and motifs: the JAZ, PPD, ZML, and TIFY; however, not all subfamilies are present in all species. For example, the TIFY subfamily is absent in *P. persica* [2] and *Camellia sinensis* [5]; the PPD subfamily is present only in dicotyledonous plants but not in monocotyledonous plants [10]. One possible reason is that the PPD subfamily of genes is more important for growth and developmental processes in monocotyledonous plants [12]. Like dicotyledons such as *P. trichocarpa* [12] and *Juglans regia* [28], *E. japonicas* has members of all four subfamilies. The homology of the *EjTIFY* genes and phylogenetic analyses with other species can help predict the potential functions of these genes [44]. Transgenic *A. thaliana* with AtJAZ1 and AtJAZ2 showed greater salt tolerance than did wild-type plants [27]. The overexpression of *OsJAZ9* in *O. sativa* can improve *O. sativa’s* tolerance to salt stress [10]. *MdJAZ15* and *MdPPD1* are upregulated under salt stress in *M. domestica* [32], so it can be hypothesized that the homologous genes *EjJAZ16* and *EjPPD1* may also affect the expression of these genes in *E. japonica* under salt stress.

In this study, the number of proteins identified from the *E. japonica* genome, 26, was greater than the TIFY proteins in *P. persica* [2], *A. thaliana* [4], *P. trichocarpa* [12], *O. sativa* [10], and *V. viniferas* [7]; however, it was less than that in *M. domestica* [11], *G. max* [26], *Manihot esculenta Crantz* [43], and *Brassica napus* [45]. Gene duplication events are the main drivers of rapid gene family expansion and evolution. In this study, there were only two pairs of tandem duplication events and 13 pairs of fragment duplication events. This typical type of low tandem frequency and high segmental duplication is consistent with the findings of existing studies [46]. Previous studies have shown that function and expression patterns of segmental repeat genes are usually similar [47, 48]. In the present study, the expression patterns of the segmental repeat gene pairs were not identical, unlike previous studies. For the *EjJAZ15*/*EjJAZ16* duplicated gene pair, under temperature stress, *EjJAZ15* was differentially and significantly expressed at both temperatures, whereas the expression of the *EjJAZ16* gene did not significantly differ. The expression patterns of the duplicate gene pairs *EjJAZ2*/*EjJAZ3* differed, while the expression patterns of the gene pairs *EjJAZ1*/*EjJAZ4* and *EjZML1*/*EjZML2* were similar. The various expression patterns among the duplicate gene pairs suggest that the gene pairs may perform different functions [49]. In addition, the strongly purified selection signal detected in the duplicated gene pairs indicates the functional importance of the *EjTIFY* genes. These results suggest that gene duplication events, especially segmental duplications, contributed to the evolution and expansion of the *TIFY* gene family in *E. japonica*.

Plant specificity is usually the result of selective loss or gain of genes during evolution, and it can involve chromosomal rearrangements and fusions, such as three typical exon/intron diversified gene duplications, exon-intron gain/loss, and exon/pseudoexon and insertion/deletion [50]. Except for *EjJAZ12*, *EjJAZ13*, and the genes in the JAZ II subclade, which contain only two exons, the other JAZ genes contain 3–7 exons; the *EjZML* subclade has 6–7 exons, except for *EjZML5* and *EjZML4*, which have 9 and 10 exons, respectively, and we can hypothesize that the reason for the multiple of these genes may be the acquisition of additional exons. The exon/intron arrangement and sequence motif structure are not complex. Motif 4 is present only in *EjZML* subfamily proteins. Most paralogous homologous pairs have similar structures, but some differences may exist. For example, *EjZML5* has nine exons, whereas the paralogous homolog *EjZML4* has ten exons, and evolution explains the existence of homologous structures adapted to different purposes as a result of evolution from a common ancestor and subsequent mutation [51]. The exon/intron and motif structures suggest that some genes may have undergone multiple rounds of chromosomal rearrangements and fusions with a tendency to lose/gain exons because of evolutionary selection with substantial changes in their structure [12].

Abiotic stresses such as low temperature, high temperature, and salt are limiting factors for plant growth and development, and proteins in the *TIFY* family play vital roles in plant stress responses, especially the JAZ subfamily, which plays a role in mediating the stress response. Proteins in the JAZ subfamily are involved in the jasmonate signaling pathway, one of the mechanisms by which plants resist environmental stimuli. JAZ proteins induced by jasmonate play a central role in the JA signaling pathway. JAZ proteins interact with COI1 in the presence of jasmonate to derepress transcription factors and activate the transcription of JA-responsive genes [3, 36]. Thus, members of the JAZ subfamily of TIFY proteins play vital roles in plant growth and development. For example, overexpression of *OsJAZ9* in *O. sativa* significantly enhanced *O. sativa* salt and drought stress tolerance [52]. Overexpression of *MdJAZ2* in *M. domestica* (*Malus domestica*) increased resistance to salt and drought treatments in *A. thaliana* [53]. The expression of *JrTIFY14*, a member of the JAZ subfamily, significantly increased under both high-temperature and salt stress and was greater than that of other genes [26]. The JAZ subfamily in *Zea mays* and *Solanum lycopersicum* responds more strongly to abiotic stress than other subfamilies [54]. The JAZ subfamily of *Populus trichocarpa* was highly expressed in different tissues under different stresses, and *PtJAZ8* was significantly expressed in all tissues. *PtJAZ6*, *PtJAZ7*, and *PtJAZ12* are highly expressed in ligustrum, xylem, and leaf tissues [12]. Seven *DhJAZ* genes were differentially expressed in *Dendrobium huoshanense* under low temperature and drought stress [55]. The JAZ subfamily also exhibited higher expression levels than did the other subfamilies under the four stresses in this study, suggesting that the JAZ subfamily plays a vital role in responding to many environmental stimuli, consistent with previous studies. According to publicly available RNA-seq data analysis, *EjJAZ* subfamily genes were expressed abundantly under different stresses. In contrast, the other TIFY subfamilies exhibited lower expression levels under stress. In addition, qRT-PCR analysis results revealed that the *JAZ* subfamil*y* genes played more vital roles than did the other *TIFY* subfamily genes in response to different stress treatments.

## Conclusion

In this study, the *EjTIFY* gene family was systematically analyzed. Twenty-six *TIFY* genes were identified in the *E. japonica* genome, including 16 in the JAZ subfamily, seven in the ZML subfamily, one in the PPD subfamily, and two in the TIFY subfamily. The phylogenetic relationships, chromosomal localization, duplication events, conserved motifs, and gene structures of these 26 *TIFY* genes were analyzed. In addition, the expression profile analysis of *EjTIFY* showed that some of the genes were regulated by adverse stresses, such as low temperature, high temperature, salt, and gibberellin treatments, suggesting that the *EjTIFY* genes play a vital role in *E. japonica* stress tolerance. In addition, *EjJAZ* genes play a regulatory role in plant growth and development under adverse conditions. *EjJAZ2*, *EjJAZ4*, and *EjJAZ9* respond to plant salt stress resistance, which may enable plants to gain resistance to salt stress through the JA pathway.

## Materials and methods

### Identification and physicochemical properties of the *TIFY* gene family in loquat

To identify *TIFY* family members from the whole-genome sequence of *E. japonica*, the genome sequence and annotation files of *E. japonica* were downloaded from CNGBd (https://db.cngb.org/search/assembly/CNA0019282/). The protein sequences of Arabidopsis, rice, peach, and apple were obtained from this article [2]. First, the TIFY Hidden Markov Model (PF06200) was acquired from the Pfam database (http://pfam-legacy.xfam.org/). Subsequently, the HMM profile was utilized to screen the loquat proteomes using the hmmsearch software in the HMMER package v3.2.0 to identify potential members of the *TIFY* gene family. The TIFY HMMER file was used as the reference sequence to filter out the E-value < 1 × 10^−5^ from the results of protein sequence numbers [56], followed by the downloading of protein sequences of *A. thaliana TIFY* gene family members from the *A. thaliana* genome database TAIR (https://www.arabidopsis.org/index.jsp). Then, the BLASTP program was used to compare the homology of the *E. japonica* genome with the TIFY protein sequences of *A. thaliana* as a reference to screen out the protein sequences with E-value > 1e^−5^, and then the HUMMER search program was subsequently used to compare the protein sequences of the *TIFY* gene family members [57]. Finally, the Conserved Domain Database (CDD) tool of the NCBI database (https://www.ncbi.nlm.nih.gov/cdd) and the online software Pfam (http://pfam.xfam.org/) were used to confirm the presence of the TIFY structural domain [58]. The sequences of nonexistent or incomplete TIFY structural domains were removed from the *E. japonica* TIFY gene family members. The physicochemical properties were analyzed using ProtParam (http://www.expasy.org/tools/protparam.html) online software for molecular weight (MW), isoelectric point (PI), amino acid number, and instability coefficient (aliphatic index) [59]. Finally, subcellular localization was performed using the Wolf Psort (https://wolfpsort.hgc.jp/) online tool.

### Gene structure and conserved motif analysis

TBtools software was used to analyze the gene structure of each gene based on the genome annotation file (gff3) of *E. japonica* [60]. The structural domains were then predicted using the CDD-search online tool (https://www.ncbi.nlm.nih.gov/cdd) from the NCBI database with default parameters [58]. The conserved motifs of TIFY proteins were analyzed using MEME (http://meme-suite.org/) online software [61] to determine the differences among the *EjTIFY* gene family members. Finally, the above results were visualized by TBtools software [60].

### Phylogenetic analysis

The protein sequences of the *TIFY* gene identified from *E. japonica* were merged with those of *A. thaliana*, *O. sativa*, *V. vinifera*, *M. domestica*, and *P. persica*, and sequence comparisons were performed using MAFFT software [62]. Sequence comparison was conducted, and a phylogenetic tree was subsequently constructed by selecting the maximum likelihood (ML) method using IQtree software [63]. Chipplot (https://www.chiplot.online/) online software was used to visualize the evolutionary tree. Finally, the 26 EjTIFY proteins were grouped according to the grouping of *A. thaliana* TIFY proteins [4].

### Analysis of promoter cis-acting elements

The 2,000 bp sequences upstream of the JAZ, PPD, TIFY, and ZML coding DNA sequences were extracted from the genome of *E. japonica* using TBtools software. The sequences were uploaded to the PlantCARE database (https://bioinformatics.psb.ugent.be/webtools/plantcare/html/) [64] for the prediction of cis-acting elements in the promoter regions of *E. japonica* JAZ, PPD, TIFY, and ZML. We referred to Longbo Liu’s method [65] and used RStudio for beautification.

### Chromosome localization and covariance analysis

The chromosomal locations of each JAZ, PPD, TIFY, and ZML member were confirmed using *E. japonica* genome annotation data. The occurrence of gene duplications was assessed by examining both tandem and segmental duplication processes during gene amplification. To understand the amplification pattern of the *EjTIFY* gene family, we analyzed the tandem duplications occurring in the TIFY gene family using the multicollinear tool MCScanX [66]. We selected the NG method to compute the nonsynonymous substitution rate (Ka), the synonymous substitution rate (Ks), and the selection pressure Ka/Ks for each pair of duplicated genes. Numerous studies have shown that two neighboring genes are considered tandem duplicates [20]; tandemly duplicated gene pairs with Ka/Ks > 1 are positively selected, and Ka/Ks = 1 are neutrally evolved; Ka/Ks < 1 is considered to be subject to purifying selection.

### Expression analysis of gibberellin-induced loquat tissues

Plant flower formation is influenced by many endogenous and exogenous factors, which form a very complex regulatory network. It can accurately respond to internal and external signals and integrate them to ensure that plants flower at a favorable time and reproduce successfully [67]. One of the vital hormones affecting plant flowering is gibberellin [68]; therefore, in this study, we downloaded the transcriptome data of *E. japonica* leaves and buds induced by gibberellin from the NCBI database according to the accession numbers in published papers, which were used to explore the vital genes affecting *E. japonica* flowering (Table 1).

**Table 1:** Sources of transcriptome data

Accession	Stress	CK/T	Time	Replicate	Resource
PRJNA623262	Low temperatures	Room temperature	24 h	3	NCBI
		-1 ℃ and − 3 ℃	24 h		
PRJNA762417	High temperatures	0 min	40 ℃	3	NCBI
		60 min and 90 min	40 ℃		
GSE249070	Salt stress	0 mg/L	10 d	3	Research group experiments
		200 mg/L	10 d		
PRJNA729650	Leaves and shoots treated with GA	0 mg/L	3 m	3	NCBI
		300 mg/L	3 m		

### Plant materials and treatments

In this paper, the seeds of *E. japonica* from Mengzi, Yunan, China, were selected, and sterile live plants were obtained via histoculture; the plants were subsequently transplanted outdoors for expansion. The plants were subjected to salt stress treatment after they grew into complete plants. In the previous research of this research group, 200 mmol/L NaCl [69] was selected to treat the loquat seedlings, which was recorded as T, and clear water was used as the control, which was recorded as CK. Three biological replicates were set up for each treatment, and the treated samples were quickly stored in a liquid nitrogen environment at -80 °C and sent to Noemi Metabolism Ltd.　(Zhejiang, Suzhou, China)　for RNA extraction. Then, the libraries were constructed for sequencing after quality inspection.

### Expression analysis of the *E. japonicaTIFY* gene under abiotic stresses

To understand whether the *EjTIFY* gene functions under various abiotic stresses and to explore its expression regulation pattern, we analyzed the *EjTIFY* gene under three abiotic stresses (salt, low temperature, and high temperature). The transcriptome data were downloaded from the NCBI database according to the accession number (Table 1).

To avoid excessive adapters and low-quality data in the transcriptome data, we used Trim-Galore and Trimmomatic software to remove adapters and filter low-quality data, used FastaQC to check the quality of the data, and finally ensured that the q-value of all the transcriptome data was greater than 30. The reads of the QC-passed transcripts were localized to the reference by the hisat2 genome (JFZ genome) [70], the reads were counted with the reference genome using the featuremts toolkit of Rsubread software, the fragments per kilobase of exon model per million mapped fragments (FPKM ) for each gene were calculated, and finally, the expression of each gene was quantified [71]. To make the gene expression estimated from different experiments comparable, we chose the most common gene expression normalization method to calculate the TPM value of each gene, which can integrate the effects of sequencing depth and gene length on read counts and normalize the expression of each gene. Based on log_2_ (TPM + 1) values, TBtools was used to map and analyze the expression patterns of *EjTIFY* gene family members under abiotic stress and in gibberellin-induced *E. japonica* tissues [60].

In addition, we used DESeq2 software for differential expression gene analysis [72]. The p-value of the differential test was corrected for multiple hypothesis testing, and the threshold value of the p-value was determined by controlling the false discovery rate (FDR). While obtaining the FDR value of the differential test, the differentially expressed multiple of the genes among different samples was calculated based on the expression of the gene (fold change). We defined genes with a fold change ≥ 2 and *p* ≤ 0.05 as differentially expressed genes (DEGs).

### GO and KEGG enrichment analysis

The protein sequences of 26 *TIFY* genes of *E. japonica* were submitted to the EggNOG-MAPPER database (http://eggnog-mapper.embl.de/) for gene functional annotation [34, 73]. The obtained results were analyzed by GO enrichment in TBtools and plotted and visualized with the online tool ChiPlot (http://www.chiplot.online/) for plotting and visualization [60].

### Validation of salt stress via qRT-PCR in loquat

The total RNA of *E. japonica* was extracted according to the instructions of the DP43 kit, and cDNA was synthesized with the FastKing RT Kit, a reverse transcription kit. The cDNA template was added to the reaction system by real-time PCR using an ABI7500 instrument. The reaction system included 10 µL of Power qPCR PreMix, 0.5 µL of forward and reverse primers, and 1 µL of diluted cDNA template. The reaction program was as follows: 95 °C for 10 min, 95 °C for 10 s, and 60 °C for 40 s, for 42 cycles. We used actin as an internal reference gene for the experiment. The results of the experiments were used to calculate the relative expression of the genes using the 2^−ΔΔCt^ method [74]. Statistical analysis was performed using SPSS, and *p*-values were calculated using independent samples t-tests [75]. All primers used are listed in the Supplementary Table S1.

### Supplementary Information

Supplementary Material 1: **Table S1.** All primers. 

Supplementary Material 2: **Table S2.** Identification of EjTIFY genes and analysis of their physicochemical properties.

Supplementary Material 3: **Table S3.** EjTIFY Duplicate Genes Pair Ka/ ks Values.

## Data Availability

RNA-Seq data under abiotic stress conditions can be found under accession numbers PRJNA623262, PRJNA762417, and PRJNA729650. RNA-Seq data under salt stress can be found under accession number GSE249070 (https://www.ncbi.nlm.nih.gov/geo/query/acc.cgi?acc=GSE249070). The RNA-Seq data are publicly available at the National Center for Biotechnology Information. The other data presented in this study are available in the Supplementary Information.

## Abbreviations

ZIM: Zinc-finger protein expressed in inflorescence meristem
JA: Jasmonic acid
GO: Gene Ontology
KEGG: Kyoto Encyclopedia of Genes and Genomes
Chr: Chromosome
DEG: Differentially expressed gene
HMM: Hidden Markov model
FDR: False Discovery Rate
Ka: Nonsynonymous
Ks: Synonymous
CDS: Coding sequences
TPM: Transcripts Per Kilobase of exon model per Million mapped reads
qRT-PCR: Quantitative real time-PCR
MW: Molecular weight
